# SARS-CoV-2 Treatment Approaches: Numerous Options, No Certainty for a Versatile Virus

**DOI:** 10.3389/fphar.2020.01224

**Published:** 2020-08-26

**Authors:** Simona Iacob, Diana Gabriela Iacob

**Affiliations:** ^1^Infectious Diseases Department, Carol Davila University of Medicine and Pharmacy, Bucharest, Romania; ^2^Infectious Diseases Department, National Institute of Infectious Diseases “Prof dr. Matei Bals”, Bucharest, Romania; ^3^Infectious Diseases Department, Bucharest Emergency University Hospital, Bucharest, Romania

**Keywords:** SARS-CoV-2, therapeutic targets, angiotensin-converting enzyme 2 receptors, endocytosis, immune response, monoclonal antibodies, vaccine, mesenchymal stem cells

## Abstract

SARS-CoV-2 is the most recent coronavirus which crossed the species barrier in 2019 and provoked a still ongoing and dangerous pandemic known as coronavirus disease 2019 (COVID-19). The SARS-CoV-2 infection has triggered an impressive amount of clinical and experimental studies to identify an effective and safe therapy to stop the pandemic spread. Hence, numerous trials and studies have scrutinized the analogies between SARS-CoV-2 and other corona viruses or the host-virus interactions and their similarities with immune system disorders. Still, the pathogenic mechanisms behind SARS-CoV-2 have been partially deciphered and the current therapies have not yet met the initial enthusiastic expectations. So far COVID-19 therapies have targeted various pathogenic mechanisms, namely the neutralization of ACE2 receptors or SARS-CoV-2 spike protein epitopes, the disruption of the endocytic pathways using hydroxychloroquine, arbidol, or anti-Janus kinase inhibitors, the inhibition of RNA-dependent RNA polymerase using nucleotide analogues such as remdesivir, immunosuppressive drugs or molecules acting on the immune response (corticoids, interferons, monoclonal antibodies against inflammatory cytokines, mesenchymal stem cells) and convalescent plasma administration together with numerous drugs with unproven effect against SARS-CoV-2 but with potential antiviral activities (antiretrovirals, antimalarial drugs, antibiotics, etc.). Nevertheless, these therapies have been associated with side effects and contradictory results. At the same time a specific SARS-CoV-2 vaccine is a long-term solution requiring clinical validation and important investments together with appropriate strategies to promote the confidence in the safety of the new vaccine. The article revises the current state of SARS-CoV-2 therapeutic options but advises towards a more cautious and individualized treatment approach centred on the clinical features, immune particularities, and the risk-benefit balance.

## Introduction

Coronaviruses (CoVs) are a large group of RNA viruses belonging to the subfamily of Coronaviridae, the family *Coronaviridae*, order *Nidovirales*. CoVs are widely spread across numerous species including humans and display a high interspecies adaptability. CoVs possess low pathogenicity associated with respiratory and enteric diseases usually of mild or medium severity. Nevertheless, three species of CoVs have been responsible for important and severe respiratory outbreaks in humans, namely the severe acute respiratory syndrome coronavirus (SARS-CoV) which lead to the 2002–2003 outbreak, the Midde East respiratory syndrome coronavirus (MERS-CoV) in 2012 and the severe acute respiratory syndrome coronavirus 2 (SARS-CoV-2) associated with the ongoing pandemic also referred to as CoV disease 2019 (COVID*-*19) (Huang et al., 2020).

The COVID-19 outbreak started in December 2019 in Wuhan China. It subsequently spread to other continents, so that on January 30^th^ the World Health Organization (WHO) issued a warning of public health emergency of international concern. This statement was followed by the declaration of a COVID-19 pandemic on the 11^th^ of March 2020. Currently, COVID-19 has been recorded in over 200 countries and territories. As of July 20^th^, 2020, the number of cases due to COVID-19 exceeded 14,000,000 cases and 600,000 deaths. The rapid spread and high infectivity of SARS-CoV-2 as well as its potentially lethal and unpredictable course has led to an extraordinary collective effort of researchers to characterize the virus and to identify efficient treatments and prophylactic alternatives. Hence, an impressive armamentarium of molecules was proposed for the SARS-CoV-2 treatment in a very short period of time and the list of molecules is still increasing. Multiple investigational drugs with potential antiviral activity, have been administered since the start of the outbreak either off-label or as part of clinical studies, through programmes of compassionate use. Nevertheless, currently, none of these therapeutic agents proved to be efficient and completely safe. Still, recently there has been a significant progress. As of June 2020, remdesivir, a nucleoside analogue has gained an authorization for emergency use in hospitalized patients with severe forms of COVID-19 in the United States and Europe, while dexamethasone is also recommended in specific situations and umifenovir, α-interferon, lopinavir/ritonavir, ribavirin, and chloroquine phosphate has been added to the Guidelines for the Diagnosis and Treatment of COVID-19 in China (Youyao and Yizhen Chen, 2020).

The article provides a critical analysis on the current anti-SARS-CoV-2 molecules, their specific target during the virus life cycle and their potential role as backbones for future therapeutic regimens if these would prove to be both efficient and safe against COVID-19.

## SARS-COV-2 Life Cycle

SARS-CoV-2 is a large, enveloped, single-stranded RNA virus with a positive-sense. It entails 16 non-structural proteins (Nsp) which are essential for viral replication and 4 other structural proteins namely the spike, membrane, envelope and nucleocapsid, which mediate the cell invasion and immune response (Alsaadi and Jones, 2019; Gordon et al., 2020). Certain structural proteins are highly conserved among coronaviruses such as the Nsp12-RNA-dependent-RNA-polymerase (RdRp) involved in viral replication, while others such as CoV-2 Spike (S)-protein, the main SARS-CoV-2 antigenic structure, express a limited genetic variability.

**Figure 1:**
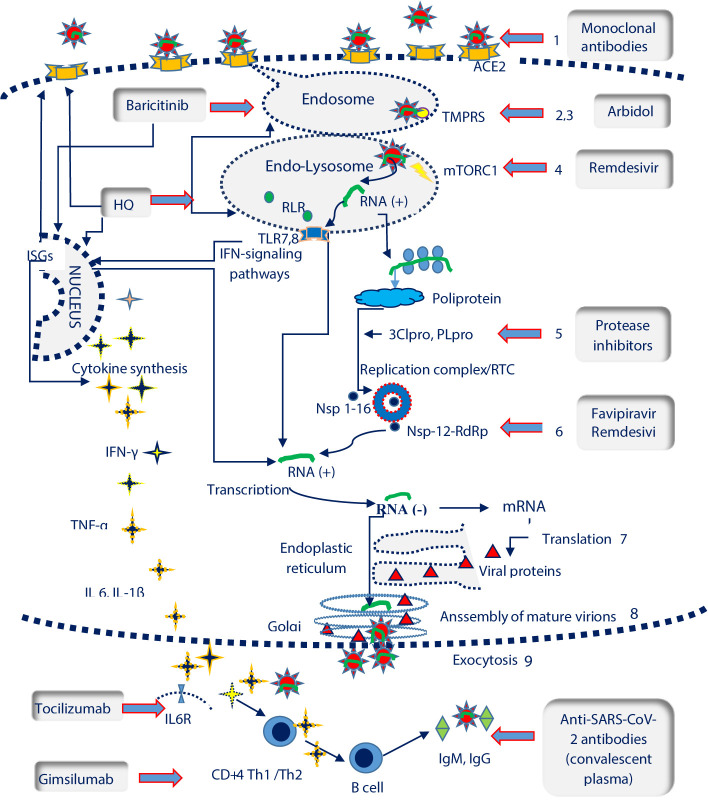
SARS-CoV-2 life cycle and potential therapeutic targets. ISGs, interferon-stimulated genes; IL6R, Interleukin 6 receptor; TLR 7,8, Toll-like receptors 7,8; TMPRSS, transmembrane protease serine 2 (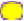); mTORC1, mammalian target of rapamycin complex 1 (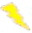); RLR, RIG-I-like receptors; ACE2, angiotensin converting enzyme 2 receptor (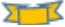); RTC, replication/transcription complex; 3CLpro, 3C-like protease; PLpro, Papain like Protease; RdRP, RNA-dependent RNA polymerase (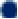); Nsp, nonstructural protein. SARS-Cov-2 life cycle and pathogenesis include: the interaction between the spike protein and ACE2 receptor on host cells (1); clathrin-mediated endocytosis (2); the fusion of the virus with cell membranes facilitated by transmembrane protease serine 2 (TMPRSS) (3); autophagy and RNA release in the presence of the mammalian target of rapamycin complex 1 protein (rapamycin complex 1) (mTORC1) (4); RNA translation , polyprotein synthesis and cleavage with the help of 3C-like protease (3CLpro) and papain like protease (PLpro) (5); genomic RNA transcription catalysed by thr RNA-dependent RNA polymerase (RdRP) in the Replication/Transcription Complex (RTC) (6); translation of viral proteins (7); assembly of viral proteins and RNA into a mature virion in the Golgi (8) ; the virion is released by exocytosis (9). Recognition of viral RNA by endosomal RIG-I-like receptors (RLRs) and Toll-like receptors (TLRs) allows the activation of type I interferon (IFNs) response, that up-regulates the expression of interferon-stimulated genes (ISGs) and activate the antiviral response. The elimination of SARS-Cov2 requires a coordinated response which should include: the cytokine release, Th1/2 immunity and synthesis of neutralizing antibodies by LB/plasma cells SARS- Cov-2 potential therapeutic targets encompass the following steps: blocking ACE2 receptors (monoclonal antibodies); TMPRSS inhibition which prevents endosomal fusion (arbidol, hidroxyclorochine); mTORC1inhibition and the controlul of autophagy and RNA transcription (remdesivir); inhibition of 2Clpro and PLpro viral proteases involved in polyprotein fragmentation (protease inhibitors namely lopinavir, darunavir, ritonavir); Nsp-12-RdRp inhibition with an essential role in the activity of the replicase RNA transcription complex (favipiravir, remdesivir); suppression of cytokine release (hydroxiclorochine, baricitinib), blocking the IL6R (tocilizumab) or Th1 response (gimsilumab); blocking the virus through the administration of neutralizing antibodies from the plasma of COVID-19 convalescents.

Cell invasion includes the viral entry steps (receptor recognition, endocytosis and viral- membrane fusion) and post-entry steps (genomic RNA translation and replication, virion assembly, maturation, and exocytosis). The host immune response is initiated as soon as the type I interferon (IFN-I) response is triggered by intracellular genomic RNA. IFN-I release leads to the transcription of hundreds of interferon-stimulated genes (ISGs) and to the recruitment of CD4 + T-helper cells, further responsible for the Th1/Th2 response and humoral immunity (**Figure 1**).

### SARS-CoV-2 Entry Steps and Therapeutic Targets

#### Receptor Recognition as a Therapeutic Target

The CoV-2 S protein is a type I transmembrane protein. It contains two ectodomains: a receptor-binding subunit (RBD) S1 which ensures the viral biding to angiotensin-converting enzyme 2 (ACE2) receptor and a membrane-fusion subunit S2 (Wan et al., 2020; Zhang et al., 2020; Zhou P. et al., 2020). The S1–S2 domain of the S-glycoprotein contains a “furin-like cleavage site” which is activated by furin, an omnipresent cellular protease (Coutard et al., 2020). Furin plays an essential role in the activation of the S protein as well as afterwards, during its cell membrane fusion and intercellular spreading (Hoffmann et al., 2020a). The activation of the S1/S2 domain enables the interaction between the S protein and ACE2 receptor followed by the downregulation of ACE2, an ubiquitous receptor preferentially expressed in epithelial or endothelial cells (Harmer et al., 2002; Kuba et al., 2005; Hoffmann et al., 2020b). ACE2 cleaves Angiotensin II (Ang II) to Angiotensin 1-7 (Ang 1-7), a vasodilatory and anti-proliferative heptapeptide. Ang (1-7) ensures the equilibrium of the renin angiotensin system and protects the vascular endothelia of the vital tissues such as brain, heart, lungs, and kidneys (Crackower et al., 2002; Harmer et al., 2002). Hence, ACE2 is at the same time a wide-spread protective enzyme and an entry receptor for SARS-CoV-2. ACE2 mRNA, has a higher expression in the respiratory epithelia, mostly in the nasal and alveolar epithelial cells but also in the epithelial cells of the salivary gland, intestinal and renal epithelia or cardiac cells (Harmer et al., 2002; Hamming et al., 2004; Sungnak et al., 2020). Circulating ACE2 levels are higher in diabetic, cardiac or obese patients especially those with organ dysfunctions or treated with angiotensin-converting enzyme inhibitors (ACEIs) and angiotensin II receptor blockers (ARBS) (Batlle et al., 2010; Yang et al., 2010; Fang et al., 2020; Nicin et al., 2020). While the correlation between the outcome of SARS and serum or tissue concentration of ACE2 or ACEIs/ARBS administration remains elusive (Albini et al., 2020; Reynolds et al., 2020), the prognosis of patients with cardiac or pulmonary disease, diabetes or obesity appears to be worse (Fang et al., 2020; Shi et al., 2020b). Hence, the protection of these key groups remains a pressing matter. Various solutions have been proposed to address the interaction between the viral proteins and ACE2, such as a recombinant soluble form of ACE2 (Khan et al., 2017; Monteil et al., 2020) or cross-reactive neutralizing antibodies active against SARS-CoV-2 RBD specific epitopes (Lan et al., 2020; Wang C. et al., 2020; Yuan et al., 2020).

#### Endocytosis and TMPRSS2 as a Therapeutic Target

After the S1 subunit attaches to the cellular receptors, the virus enters the cell through clathrin-mediated endocytosis and is then transported to the early endosomes (Inoue et al., 2007). From this point forward, the transmembrane protease serine 2 (TMPRSS2) cleaves the SARS-CoV-2 S protein for a second time and facilitates cell entry and the fusion between the endosomal membrane and the viral S2 sequence (Hoffmann et al., 2020b). Consequently, the inhibition of TMPRSS2 could prevent SARS-CoV-2 cellular entry and could play a potential therapeutic role as has been already shown in other viral respiratory syndromes and experimental models (Kawase et al., 2012; Yamamoto et al., 2016; Hoffmann et al., 2020b).

The endocytic pathway represents a key mechanism behind viral entry. Multiple studies have currently approached the antiviral role of endosomal-tropic agents such as hydroxychloroquine sulfate or umifenovir (arbidol) (Yang and Shen, 2020). SARS-CoV-2 virions are processed through the endo-lysosomal network, where the autophagy machinery is involved in the delivery of the viral genome to the host cell. The autophagy is initiated by a complex enzymatic system, also controlled by the mammalian target of rapamycin complex 1 (mTORC1). While it is still unclear how the autophagy process influences the replication of SARS-CoV-2 (Yang and Shen, 2020), mTORC1 is the target of various molecules such as remdesivir, as well as of other FDA drugs which have been approved for oncologic purposes (sirolimus) but currently unexplored in COVID-19.

### SARS-CoV-2 Post Entry Steps and Therapeutic Targets

The viral invasion of the target cells implies genomic RNA translation and the synthesis of polyproteins which are further cleaved by two viral proteases, namely 3C-like protease (3CLpro) and Papain Like protease (PLpro), in order to mature the Nsp (Macchiagodena et al., 2020). Certain protease inhibitors frequently used in the treatment of HIV such as lopinavir/ritonavir have already been tested to verify their ability to block these proteases but showed a debatable efficiency (RECOVERY trial). Viral RNA synthesis involves two stages: genome replication and subgenomic mRNAs transcription, both of which mediated by the replication/transcription complex (RTC), a membrane-bound structure encoded by the virus. Of these two processes, Nsp12-RdRp plays a central role and is targeted by various experimental molecules (favipiravir). The final stages of the viral cycle cover the viral translation of the structural proteins, the assembly of all viral components into the endoplasmic reticulum, the recreation of the virions in the Golgi apparatus and their elimination through exocytosis (de Haan and Rottier, 2005). Currently, none of these stages if targeted by specific drugs.

### Host Immune Response to SARS-CoV-2 Infection and Therapeutic Targets

SARS-CoV-2 triggers the innate immune response immediately after the endosomal receptors recognize the viral RNA. Consequently, Toll-like receptors 7/8 and RIG-I-like receptors RIG-I/MDA-5 activate transcription factors and trigger a type I IFN response. In turn, the IFN-I response stimulate infected cells to control the early stage of the viral infection through the release of proinflammatory cytokines and Th1 immune response stimulation. An imbalance of the innate immune response can potentially trigger an uncontrolled release of pro-inflammatory cytokines which accompanies the acute respiratory distress syndrome (ARDS) or other lethal organ failures (Chen et al., 2010; Conti et al., 2020) quite similar to that observed in septic patients (Li et al., 2020). Hence, the cytokine mediated storm is a significant complication of SARS-CoV-2 and has been targeted by various investigational monoclonal antibodies (mAbs) (Kewan et al., 2020).

The generation of anti-nucleoprotein IgG/IgM and anti-RBD IgG/IgM neutralizing antibody (To et al., 2020) play an undefined role to control the infection. The transfer of these antibodies from previously infected patients to new cases (plasma convalescent administration) was initially seen as a solution for severe COVID-19 cases and has been received with enthusiasm. In March 2020, the FDA has therefore granted the clinicians the permission to access and use convalescent plasma for the treatment of life-threatening SARS-CoV-2 infection. However, there is still, limited data on the sero-conversion following the SARS-CoV-2 infection as well as on its associated side effects, such as the risk of acute lung injury (Liu et al., 2019).

Consequently, the synthesis of a vaccine remains the most certain solution to ensure a direct protection against SARS-CoV-2 infection. The development of vaccines for human use represents a long-term solution, while other technologies and drug targets remain to be evaluated in the following years. While waiting for a vaccine, experimental drug therapies remain a crucial alternative in severe forms.

Detailed below are therapeutic aspects of currently available drugs and their action mechanism.

## SARS-COV-2 Experimental Drugs

The list of experimental drugs against SARS-CoV-2 has been continuously increasing with the emergence of new data on the structure and pathogenic mechanisms of the new virus. Most of the current therapeutic data has been extrapolated from the research performed on either SARS-CoV or murine hepatitis virus (MHV), a prototypic member of the CoV family (Fung et al., 2020). The available therapeutic agents target the pathogenic steps of infection (viral entry or post-entry steps) as well as the unbalance of the immune response (**Figure 1**).

### Drugs Targeting Viral Entry Steps

#### Receptor Neutralization

Currently, there have been various approaches to address ACE2-mediated SARS-CoV-2 entry. A therapeutic strategy to be taken into consideration is to prevent the virus/ACE2 attachment through mAbs specifically designed against specific epitopes of the S protein, especially the RBD-S1 unit (Sui et al., 2004; Elshabrawy et al., 2012). This unit shares a relatively high homology (73%) between SARS-CoV-2 and SARS-CoV, allowing the identification of cross reactive antibodies *in vitro* or in animal models (Tian et al., 2020).

Additionally, 2 other clinical trials were launched in June 2020, namely a phase 1 trial for the study of LyCOV555 and JS016 respectively, two neutralizing IgG1 mAbs directed again the S protein (NCT04427501, NCT04441918). It is probable that a cocktail of mAB against different epitopes could significantly improve the affinity and neutralizing activity and could lower the risk of resistance mutation. Still, in this case, the use of mAb would require an early administration so as to precede viral replication, a step which would considerably limit their practical use, along with the consideration for the development for specific technologies and the high cost. Additionally, further research is needed to clarify the potential risk related to antibody-dependent enhancement, a form of immune enhancement accompanied by cytokine storm which ensues as a result of prior exposure to other viruses or coronaviruses (Cegolon et al., 2020). SARS-CoV-2 also uses other co-receptors to infect host cells (Wang K. et al., 2020). In this respect, meplazumab, an anti-CD147 mAb is undergoing a clinical trial (NCT04275245) to verify its ability to inhibit the interaction between SARS-CoV-2 spike protein and the CD147 co-receptor.

Another therapeutic alternative would be to replete the pool of ACE2 receptors using human recombinant soluble ACE2 enzyme (hrACE2). HrACE2 was experimentally used by Monteil to block SARS-CoV-2 in vessel organoids and in human kidney organoids (Monteil et al., 2020). Furthermore rhACE2 was also used in the treatment of ARDS as an alternative to prevent both the viral entry as well as the excessive inflammatory response driven by the release of IL-6 (Khan et al., 2017; Zhang et al., 2020). Nevertheless, the inhibitory effect of rhACE2 against SARS-CoV-2 remains unknown. A pilot study on the matter has been withdrawn (NCT04287686, China) but another pilot trial using APN01, (a synonymous for rhACE2) is still ongoing (NCT04335136) and is expected to report its preliminary conclusions in September 2020. Additionally, a recombinant bacterial derived ACE2-like enzyme named B38-CAP will be evaluated in a randomized control trial regarding its ability to prevent lung injury (NCT04375046). According to previous data, the B38-CAP enzyme identified in *Paenibacillus* sp. B38 and produced through bacterial engineering shares a structural similarity to mammalian ACE2 and preserves the cardiac protective role of the renin-angiotensin system (Minato et al., 2020). Nevertheless, the impact of therapies which manipulate these receptors is unclear, as the ACE2 receptors are prominent regulators of renin-angiotensin pathway and are the ultimate target of multiple hypotensive drugs (Albini et al., 2020).

#### Endocytosis Inhibition

##### Hydroxychloroquine, a Controversial Drug

The endocytic entry of SARS-CoV-2 through a clathrin-mediated pathway as well as its fusion mechanism with the cell membrane led to the repurposing of previously known drugs such as cloroquine (Cq) and hydroxychloroquine (Hq). Chloroquine phosphate, is an antimalarial drug which acts through complex mechanisms to alter ACE2 glycosylation and endosomal pH and which ultimately prevents the cleavage of the S protein, and the attachment and viral fusion (Savarino et al., 2003; Vincent et al., 2005). Cq is currently studied in multiple clinical trials, either in monotherapy (ChiCTR2000029609) or in combination with other drugs such as remdesivir (Wang M. et al., 2020). Similarly, Hq sulfate (a less toxic derivate of Cq*)* also displays an activity spectrum which includes coronaviruses, in addition to its immunomodulatory potential through the inhibition of TLR7, 8 signalling and the suppression of TNF-α and IL6 synthesis (Picot et al., 1993; Jang et al., 2006; Bender et al., 2020). These therapeutic molecules showed an additional effect during both the entry and post-entry stages of SARS-CoV-2, as was also shown in Vero E6 cells, and were included in March 2020 in the Chinese guidelines for the management of COVID-19 (Vincent et al., 2005; Yao et al., 2020).

Nevertheless, the clinical efficiency of Cq and Hq is still unclear and the risk of side effects remains high, prompting a more reserved attitude, despite the wide optimism for the molecules (Juurlink, 2020). Thus, beginning with April 2020 numerous authors have expressed their concern regarding the side effects of Hq, especially in association with other toxic drugs or in high doses or for long-term use (Borba et al., 2020; Lane et al., 2020). In a large observational study on 1,446 severely ill patients Geleris et al, did not find a significant increase of side effects following the standard 5-days therapeutic regimens with Hq (Geleris et al., 2020). Nevertheless, as of June the 15^th^ the FDA issued a safety alert regarding the secondary arrhythmias reported in various studies and on the 18^th^ of June the Hq arm of a large international study (Solidarity Trial ISRCTN83971151,WHO) was stopped and other similar trials were withdrawn (NCT04347512, France and NCT04371926, United States). Finally, on July 1st the FDA cautioned against the use of Hq and Cq in COVID-19 due to safety issues. On the other hand, the therapeutic benefit of this regimen is still debatable. The study by Geleris et al. did not find a statistically significant correlation between this regimen and either intubation or death. On a similar note, two completed studies carried in China (NCT04261517, ChiCTR2000029868) and the preliminary data from other trials (NCT04347993, US, NCT04381936 UK RECOVERY Trial) (NCT04308668 US, Canada) also failed to confirm a therapeutic or a prophylactic benefit of Hq. There are still multiple ongoing studies which could shed more light on the matter, including NCT04315948-DisCoVeRy trial France, ChiCTR2000029803-China, NCT04334148-US, and NCT04304053-Spain. Overall, the antimalarial drugs Hq and Cq recently promoted in various clinical trials and initially considered as a promising option in COVID-19 have since became more and more debated (Shamshirian et al., 2020; Tu et al., 2020).

##### Other Pharmacologic Molecules

Chlorpromazine, a drug not yet used in the treatment of coronaviruses despite experimental results (Yang and Shen, 2020) is able to inhibit clathrin-dependent endocytosis. Baricitinib, an anti-Janus kinase inhibitor used as an anti-inflammatory drug in rheumatoid arthritis could regulate clarthrin-mediated endoctytosis and inhibit the release of cytokines (Richardson et al., 2020; Stebbing et al., 2020). In this respect, baricitinib entered in May a phase 3 of a clinical trial on the treatment of symptomatic COVID-19 in combination with either Lopinavir/Ritonavir (NCT04320277) or remdesivir (NCT04401579). Also, ruxolitinib, another anti-Janus kinase inhibitor is an immunomodulator currently approved by the FDA for treatment of polycythemia vera is currently studied in a Phase 3 clinical trial in patients with COVID-19–associated cytokine storm (NCT04362137, RUXOVID). Despite the anti-inflammatory effect (Yeleswaram et al., 2020), the side effects and safety profile of ruloxitinib are still unclear (Gaspari et al., 2020).

Endosomal fusion can be enzymatically inhibited by certain molecules through mechanisms which are not well understood. Such an example is umifenovir (arbidol), a broad-spectrum antiviral agent used in the treatment of COVID-19 patients across China, Russia, as well as in studies performed in Iran or India (IRCT20151227025726N15), with controversial efficiency (Deng et al., 2020; Lian et al., 2020). Other antivirals with a similar action mechanism are as tamifene and nafmostat, approved by FDA for other non-viral indications. Additionally, camostat mesylate, a protease inhibitor approved by the FDA in the treatment of chronic pancreatitis can also block the cellular entry of SARS-CoV-2 by inhibiting ACE2 and TMPRSS2 (Hoffmann et al., 2020b) (ongoing trial NCT04321096).

### Drugs Targeting Post-Entry Steps

SARS-CoV-2 RNA polymerase (Nsp12-RdRp) mediates the replicase/transcription complex (RTC) activity and catalyzes RNA-templated RNA synthesis (Wu et al., 2020a). Therefore RdRp, a key enzyme of RNA viruses with a pivotal role in viral replication and mutagenesis becomes a target for the design of anti SARS-CoV-2 drugs. Among these, two investigational nucleosidic analogues, namely favipiravir and remdesivir are currently in clinical studies, yet can be obtained through a compassionate programme. Favipiravir is an inhibitor of RdRp with a wide antiviral spectrum, approved for manufacture and sale in Japan and used against influenza viruses. Favipiravir displays an *in vitro* activity against SARS-CoV-2 and has currently entered numerous studies against SARS-CoV-2 in China and Japan, (ChiCTR2000030254, ChiCTR2000029544 and ChiCTR2000029600) (Coomes and Haghbayan, 2020). Remdesivir is an adenosine analogue which also controls mTORC1 signalling and has a wide spectrum against coronaviruses including resistant strains (Sheahan et al., 2017; Agostini et al., 2018). Remdesivir has shown *in vitro* activity against SARS-CoV-2 in combination with chloroquine (Wang M. et al., 2020) or in monotherapy, as part of a 10-day intravenous treatment in COVID-19 patients with severe pneumonia (Adaptive COVID-19 Treatment Trial ACTT-NCT04280705) (Beigel et al., 2020). Moreover, the intravenous use of remdesivir on a compassionate-use basis to patients with SARS-CoV-2 infection receiving oxygen support, has been associated with a clinical improvement in 68% of patients, according to a study published in April by Grein et al. (2020). Still, the afore-mentioned study lacked a randomized group and did not control for the viral load, aspects which have stemmed various discussions. The authors have since acknowledged some errors regarding their conclusions and have corrected their article. A large Phase 3 trial has been launched by Gilead to investigate the role of safety and efficiency of remdesivir administered for 5 or 10 days in patients with severe COVID-19 and pneumonia (NCT04292899). The partial results of these study published in May could not assess the real benefit of remdesivir (Goldman et al., 2020). Furthermore, another study from April 2020 by Wang et al. (NCT04257656) failed to provide convincing evidence regarding a significant clinical or antiviral effect of intravenous remdesivir in critical patients (Wang Y. et al., 2020). Nevertheless, the recent results from the phase 3 SIMPLE trial in hospitalized patients with moderate COVID-19 pneumonia recommends the use of Remdesivir early in the course of the disease. Additionally, the preliminary data analysis of the ACTT trial prompted an emergency use authorization (EUA) for remdesivir in the US. At the same time, the ACTT trial also enabled the authorization of remdesivir in Europe, beginning with June 2020, for patients with pneumonia requiring supplemental oxygen.

Remdesivir is thus the second major drug against COVID-19 with promising results according to the studies provided by Gilead, but which still awaits a definite confirmation. An inhalatory administration of remdesivir is going expected to be evaluated in a clinical trial beginning with August 2020, while other multiple clinical trials are examining the intravenous administration of remdesivir (WHO Solidarity trial, DisCoVeRy trial-France, ACTT-II-US).

Other nucleosidic analogues were recently disproved by clinical studies. These include ribavirin (ChiCTR200002938) and protease inhibitors targeting PLpro and 3CLpro, such as darunavir/ritonavir (Phase 3 NCT04252274) and lopinavir/ritonavir (Phase 3 NCT04251871, NCT04255017, ChiCTR2000029539 NCT04252274, ChiCTR2000029308, NCT04295551). Moreover, the latter studies on protease inhibitors received negative reviews even before the ending of these studies (Cao et al., 2020; Meyer et al., 2020). The translation of certain non-structural proteins can be inhibited by antibiotics targeting ribosome protein synthesis, such as the group of cyclines (Wu et al., 2020a), as well as macrolides (azithromycin) who also inhibit cytochrome P450. Nevertheless, there are no convincing clinical trials on this matter. Furthermore, the administration of azithromycin also poses multiple cardiovascular risks (Ray et al., 2012) and the optimistic results published by Gautret et colab (Gautret et al., 2020) on the association between Hq and azithromycin (NCR04321278) have been questioned by the International Society of antimicrobial chemotherapy on April 3^rd^ 2020 (Voss, 2020).

### Therapies Which Target Host Antiviral Defence

#### Interferons

The pathogenesis and the outcome of COVID-19 are closely related to the host immune response. The viral RNA recognition by the intracellular receptors activates IFN-I signalling pathways involved in the regulation of the inflammatory and antiviral response. A delayed or attenuated IFN-I response has been recorded in SARS-CoV-2 infections complicated with systemic inflammatory response and acute respiratory distress syndrome (ARDS) (Li et al., 2020; Jose and Manuel, 2020; Prompetchara et al., 2020). The induction of IFNs and the transcription of the interferon stimulated genes (ISGs) triggers specific antiviral pathways which directly target the viral transcriptome and proteome activity. The interaction between the ISGs and viral RNA has been better explored in other viral infections (Goraya et al., 2020) but has been less documented in SARS-CoV-2 infection. Recently, Ziegler et al. proved that ACE2 SARS-CoV-2 receptor is an ISG found in human airway epithelial cells. The authors have thus identified a surprising alternative which allows the virus to hijack the innate immunity to attenuate the immune response and to entry the human cells (Ziegler et al., 2020). Currently, it is not well understood how the virus triggers an IFN-type I response, not how it influences the pathogenesis of COVID-19. In this respect, it is unclear whether is stimulates a protective response similar to MERS-CoV (Fung et al., 2020) or an excessive inflammatory response (Channappanavar et al., 2016; Channappanavar et al., 2019) as seen in SARS-CoV-infected mice. It is possible that IFN-type I response plays a protective role in the initial phase of the infection but later contributes to the “cytokine storm’’ either directly or through the induction of other cytokines, as was proved during the MERS infection (Channappanavar et al., 2019). Hence, IFN therapy could play a pivotal role in COVID-19 clinical improvement and viral load lasting according to a recent study (NCT04276688) (Hung et al., 2020). Still, the previous study was disputed as a result of the patient selection criteria and the timing of IFN administration. Additionally, the control group received protease inhibitors, known to display a limited efficacy (Gandhi, 2020). Another notable issue on the subject is the type of IFN which efficiently inhibits SARS-CoV-2. On this matter, even though IFNβ1 displays an anti-inflammatory role on the pulmonary endothelia, it does not appear to reduce the mortality of ARDS (Ranieri et al., 2020).

Data from ongoing studies (**Table 1**) is expected to clarify the best timing, best IFN and best benefit compared with other molecules directed against SARS-CoV-2. In the meantime, IFN-based therapies in SARS-CoV-2 administered through aerosols (Sallard et al., 2020) (ChiCTR2000029638) or subcutaneously (IFNβ1b-NCT04276688/IFNβ1a-NCT04315948) should be closely supervised and require a very careful interpretation. Presently, various types of IFN are currently investigated in over 52 clinical trials in COVID-19 patients according to the Cochrane database.

**Table 1:** Interferon therapy in clinical evaluation in SARS-CoV-2 infection (July 20, 2020).

**Biological treatment**	**Study identifier number, locations**
IFN-beta, cholchicine, rivaroxaban, aspirin	NCT04331899, Canada
IFN-beta, remdesivir, chloroquine, hydroxychloroquine, lopinavir/ritonavir	ISRCTN83971151 (Solidarity), WHO, multi-country clinical trial
IFN-beta1a, hydroxychloroquine, lopinavir/ritonavir, remdesivir	NCT04315948 (DisCoVeRy), multi-centre (Europe)
IFN-beta1a	NCT04385095, UK
IFN-beta1a, lopinavir, ritonavir, hydroxychloroquine	NCT04350671 (IB1aIC), Iran
IFN-beta1a, lopinavir, ritonavir, hydroxychloroquine	NCT04350684 (UAIIC), Iran
IFN-beta1b, hydroxychloroquine	NCT04350281, Hong Kong
IFN-alfa (aerosols), lopinavir/ritonavir, traditional Chinese medicines	NCT04251871, China
Recombinant human IFN-alpha1b	ChiCTR2000030480, China
IFN-alpha aerosol inhalation, lopinavir	ChiCTR2000030535, China
IFN-alpha, ribavirin, lopinavir/ritonavir	ChiCTR2000029387, China
IFN-alpha atomization, lopinavir/ritonavir, favipiravir	ChiCTR2000029600, China
IFN-alpha, traditional Chinese medicine	ChiCTR2000029756, China
IFN-alpha aerosol/lopinavir/ritonavir	ChiCTR2000031196, China
IFN-alpha2b, tocilizumab, losartan, remdesivir, hydroxychloroquine + combinations, methylprednisolone, convalescent serum	NCT04349410 (FMTVDM), SUA
Recombinant human IFN-alpha 1b	ChiCTR2000030922, China
Recombinant human IFN-alpha-1b (nasal drops)/thymosin alpha 1	NCT04320238, China
PegIFN-α2b atomization, arbidol hydrochloride	NCT04254874, China
Long-acting IFN-alpha2a plus ribavirin	ChiCTR2000030922, China
PegIFN-lambda-1a	NCT04331899, SUA
PegIFN-lambda1a	NCT04354259 (ILIAD), Canada
PegIFN-lambda1a	NCT04344600, (PROTECT), SUA
HeberFERON (recombinant IFN-α2b and IFN-γ, Heberon alfa (IFN-alfa2b)	RPCEC00000307, Cuba

Resources: U.S. National Library of Medicine https://clinicaltrials.gov, International Clinical Trials Registry.

Platform (ICTRP) https://www.who.int/ictrp/en/, Cochrane database (https://covid-nma.com/treatment_mapping/), Chinese Clinical Trial Registry (http://www.chictr.org).

#### Monoclonal Antibodies

SARS-CoV-2 is one of the most potent viruses to initiate the sepsis cascade and to induce pulmonary lesions with the development of ARDS (Lin et al., 2018; Shi et al., 2020a). The upregulation of pro-inflammatory cytokines and especially of IL-6 was observed in all severe forms, correlated with the incidence of the plasma viral load (Chen et al., 2020) or other endogenic factors such as diabetes, cardiovascular disease or their associated treatments (Qu et al., 2014). Antiinflammatory and immunosuppressive drugs acting in monotherapy or in combination could potentially alleviate pulmonary inflammation and could be indicated in severe respiratory forms such as ARDS. Such examples include tocilizumab, a humanized IgG1 anti IL-6 receptor mAb commonly used in the treatment of rheumatoid arthritis and also approved in the treatment of cytokine release syndrome. Tocilizumab has already reached multiple Phase 2 (NCT04317092, NCT04315480) and phase 3 trials (COVACTA, NCT04320615) and preliminary data are encouraging (Xu et al., 2020). In China, tocilizumab has already been approved in COVID-19 critical patients with severe respiratory failure. Other mAb which could mediate the release of cytokines in patients with severe/critical COVID-19 lung injury include the anti-IL6 receptor mAbs sarilumab and siltuximab (NCT04324073, NCT04315298, NCT04329650) or anti-VEGFA mAb bevacizumab (NCT04275414). Gimsilumab, a human monoclonal antibody targeting Granulocyte Macrophage-Colony Stimulating Factor (GM-CSF) is the most recent monoclonal antibody proposed for the treatment of COVID-19 and will be assessed through the US BREATH clinical trial for the ARDS induced by SARS-CoV2 (Zhou Y. et al., 2020). GM-CSF is a myelopoietic growth factor with an immunomodulatory role for which it is used in autoimmune diseases. Although GM-CSF plays a central role to coordinate the cytokines which intervene in the inflammatory tissue distruction (Bhattacharya et al., 2015), there is no data to support that this process also occurs during the SARS-CoV-2 inflammatory reaction. Taking into account the complex role of GM-CSF (Zhan et al., 2019), therapeutic trials on GM-CSF require a solid theoretic basis and unfortunately preliminary results are not expected in a close future. The same argument can be made with other clinical trials using immunomodulatory drugs with scarce data on their efficiency in viral or autoimune diseases, such as thalidomide (NCT04273529, NCT04273581) or fingolimod (NCT04280588). Additionally, experimental data on the efficiency of these molecules in COVID-19 are missing.

#### Anti-Inflammatory Drugs

Data on anti-inflammatory drugs has been contradictory in what concerns their immunosuppressant effect such is the case of corticosteroids or their potential to increase the ACE2 expression such is the case of ibuprofen. Nevertheless, ciclesonide, an inhalatory corticosteroid, has been experimentally shown to suppress SARS-CoV-2 growth and host inflammation in the lungs and some published cases has confirmed this aspect (Iwabuchi et al., 2020; Matsuyama et al., 2020). Recent studies have advocated for the therapeutic benefit of corticosteroids in critically ill adults with COVID-19 (Horby et al., 2020; Wu et al., 2020b). The investigators of a large clinical trial supported by the Oxford University (UK RECOVERY trial NCT04381936) have reported a positive effect of intravenous dexamethasone administration in the treatment of COVID-19. The study showed that a 10-day treatment with low doses of dexamethasone (6 mg/day) reduced the mortality in patients who required oxygen support. Still, the authors underlined that this positive effect was not recorded in patients who did not require oxygen. This observation additionally highlights the need to adapt the treatment to the dynamic nature of COVID-19 and to adopt differential strategies between the early viral invasion stage and the late stage dominated by a strong inflammatory response.

#### Cell Therapy

The pathogenicity of coronaviruses is highly correlated with the immune dysregulation and the inflammatory response that is triggered by viral particles in the respiratory tract (Channappanavar and Perlman, 2017; Huang et al., 2020). Hence, various authors have proposed the therapeutic use of mesenchymal stem cells (MSC) known for their broad immunomodulatory and multipotent differentiating features.

MSC migrate towards the affected lung tissue and induce the release of antimicrobial peptides such as LL37 (Krasnodembskaya et al., 2010), which in turn attenuate the inflammation and promote the regeneration of damaged cell (Shi et al., 2018). MSC are cells with a marked functional plasticity, which can either promote or inhibit the immune response depending on the local inflammatory status (Spaeth et al., 2008). Currently, MSC have been successfully used for immune disorder therapy and graft-versus-host disease (Aggarwal and Pittenger, 2005). Even though the impact of MSC therapy on the outcome and mortality of ARDS varies and is still unclear (Cruz and Rocco, 2019), MSC are still considered a promising candidate in the study of SARS-CoV-2 associated ARDS (**Table 2**). According to Leng et al. (2020) the intravenous administration of MSC in patients with severe COVID-19 pneumonia shifts the immune response from a Th1 to a Th2 response, controls the inflammatory cytokines and modifies the active status of dendritic cells and macrophages. In a clinical setting, this translates to an attenuation of the respiratory failure after 2–4 days (Qu et al., 2020). The administration of MSC proved safe, with no side effects even for doses exceeding 3 times the normal dose (Bellingan et al., 2019; Qu et al., 2020). Despite these encouraging advances, no statistically significant benefit was reported for MSC treatment, probably also due to the heterogenous selection criteria of these studies. The administration of MSC through a compassionate use program, could be beneficial for critical cases of COVID-19 and could shed more light on the role of MSC.

**Table 2:** Cellular therapies in clinical evaluation in SARS-Cov2 infection (July 20, 2020).

**Biological product**	**Study identifier number, country**
Allogeneic adult MSC of expanded adipose tissue	NCT04366323, Spain
Allogenic and expanded adipose tissue-derived MSC	NCT04416139, Mexico
Adipose-derived MSC (HB-adMSCs)	NCT04348435, SUA
Autologous adipose-derived MSC (HB-adMSCs)	NCT04349631, SUA
Allogenic pooled olfactory mucosa-derived MSC	NCT04382547, Belarus
Placental MSC exosomes	ISRCTN33578935, Germany
Umbilical cord-derived MSC	NCT04333368, France
Umbilical cord-derived MSC	NCT04366271, Spain
Umbilical cord-derived MSC (hucMSCs)	ChiCTR2000030300, China
Umbilical cord-derived MSC (UC-MSC)	NCT04355728, SUA
Human umbilical cord-derived MSC	ChiCTR2000030116, China
Human umbilical cord-derived MSC (UC-MSCs)	NCT04269525, China
Umbilical cord blood mononuclear cells	ChiCTR2000029572, China
Human embryonic-stem cells derived M cells (CAStem)	NCT04331613, China
Human embryonic- stem cells derived M cells (CAStem)	ChiCTR2000031139, China
Wharton’s Jelly MSC (derived from cord tissue of newborns)	NCT04313322, Jordan
Human amniotic fluid	NCT04319731, SUA
MSC (remestemcel-L)	NCT04371393, SUA
MSC	NCT04366063, Iran
MSC	NCT04361942, COVID_MSV, Spain
MSC	ChiCTR2000030020, China
MSC	NCT04252118, China
MSC	NCT04339660 (iunie), China
MultiStem^®^ Therapy	NCT04367077, MACoVIA, SUA
MSC (called PLX-PAD)	NCT04389450 (140), SUA
MSC in combination with ruxolitinib	ChiCTR2000029580, China
Allogeneic human dental pulp derived-stem cells	NCT04336254, China
Natural Killer Cells	NCT04280224, China
FT516 (pluripotent stem cell derived NK cells)	NCT04363346, SUA
CYNK-001 (Natural Killer cells derived from human placental CD34+ cells)	NCT04365101, SUA
M1 (type I macrophage) inhibition therapy	ChiCTR2000029431, China

Mesenchymal stem cells, MSC.

Resources: U.S. National Library of Medicine https://clinicaltrials.gov, International Clinical Trials Registry Platform (ICTRP) https://www.who.int/ictrp/en/, Cochrane database (https://covid-nma.com/treatment_mapping/ ), Chinese Clinical Trial Registry ( http://www.chictr.org ).

#### Convalescent Plasma

Antibodies from convalescent plasma of COVID-19 patients can favour clinical remission and help reach temporary immunity (Rogers et al., 2020; Zhou and Zhao, 2020). Currently WHO ICTRP and Clinical Trials.gov database has recorded 51 studies on convalescent plasma, (Recovery, NCT04381936, NCT04373460, ISRCTN50189673, NCT04348656, etc. There are several caveats to this alternative, mostly related to the variable humoral response (Wu F. et al., 2020) or to the absence of certain information regarding the effective dosing size or the administration regimen, as well as the side effects. Convalescent plasma has already been used in critical COVID-19 patients through compassionate care programmes and as of April 2020, this treatment is considered an “emergency investigational new drug” authorized by FDA in clinical trials. The benefits of convalescent plasma have been reported in small COVID-19 study groups only, so that presently there is only limited data on this therapy (Bloch et al., 2020). Side effects of this treatment could exclude critical patients from the clinical trials (Li et al., 2011). In this case the high risk of transfusion associated circulatory overload restricts or even contraindicates the administration of plasma in ARDS or in cardiac patients, highlighting the need for well-documented RCTs on the matter (Food and Drug Administration (CBER), 2018; Rygård et al., 2018). In this respect, a recent assessment on the administration of convalescent plasma in COVID-19 patients has raised a series of question marks related to its safety and efficacy (Piechotta et al., 2020).

## Vaccines

The rapid spread of the COVID-19 pandemic has prompted the development of technological platforms adapted to this new pathogen and which could be employed to manufacture a safe and efficient vaccine. Currently, the World Health Organisation’s draft landscape of COVID-19 vaccines has recorded over 100 SARS-CoV-2 candidate vaccines worldwide. Most of these candidate vaccines are undergoing preclinical evaluation, while 19 have already reached clinical evaluation. Of these, 14 vaccines are specifically based on the S protein (**Table 3**).

**Table 3:** SARS-CoV-2 candidate vaccines in clinical evaluation (July 20, 2020).

**Vaccine, sponsor/enrollment age**	**Study identifier number (phase, country/estimated first results)**
**Vector-based vaccines**	
ChAdOx1 nCoV-19 (Oxford University); genetically altered adenovirus expressing the SARS-CoV-2 spike protein/18 to 55 years	ISRCTN89951424 (ph 3, Brazil)/July, 2021NCT04324606 (ph 1-2, United Kingdom)/May, 2021
Ad5-nCoV (Beijing Institute of Biotechnology, CanSino Biologics Inc); adenovirus type-5 vectored vaccine expressing SARS-CoV-2 full-length spike protein/18 to 60 years	NCT04341389 (ph 2, China)/January, 2021NCT04313127 (ph 1, China)/December, 2020
Gam-COVID-Vac (Gamaleya Research Institute, Health Ministry of the Russian Federation); adenoviral-based vaccine/18 to 60 years	NCT04436471 (ph 1, Russian Federation/August, 2020
Gam-COVID-Vac Lyo (Gamaleya Research Institute, Health Ministry of the Russian Federation/Accellena Research); lyophilizate adenoviral-based vaccine/18 to 60 years	NCT04437875 (Phase 1-2, Russian Federation/August, 2020
LV-SMENP-DC vaccine (Shenzhen Geno-Immune Medical Institute); lentiviral vector system (NHP/TYF) expressing viral proteins and immune modulatory genes to modify dendritic cells (DCs) and to activate T cells/6 months to 80 years	NCT04276896 (DC vaccine + LV-SMENP-DC) (ph 1-2, China)/July, 2023
SARS-CoV-2 aAPC Vaccine (Shenzhen Geno-Immune Medical Institute); NHP/TYF expressing viral proteins and modified artificial antigen-presenting cell (APC) to reactivate T cells/6 months to 80 years	NCT04299724 (ph 1, China) /July, 2023
**Nucleic acid vaccines**	
mRNA-1273 (NIAID); lipid nanoparticle (LNP)-encapsulated mRNA-based vaccine encoding full-length, spike protein of SARS-CoV-2/18 to 99 years	NCT04283461 (ph 1, SUA) /November, 2021
mRNA-1273 (mRNA-1273 + Placebo) (Moderna TX Inc, Biomedical Advanced Research and Development Authority); two dose levels of mRNA-1273 versus placebo/18 years and older	NCT04405076 (ph 2-3, SUA) /March, 2021
BNT162, (BioNTech Pharmaceuticals GmbH); Dose-Escalation Trial **of** four RNA vaccine candidates combined with a lipid nanoparticle formulation (LNP) (different dosing regimens)/18 to 55 years	NCT04380701 (ph 1-2, Germany) /August, 2020
BNT162 (BioNTech, Pfizer); LNP-mRNAs (different dosing regimens)/18 to 85 years	NCT04368728 (ph 1-2, SUA, Germany)/June, 2021
INO-4800 + electroporation using CELLECTRA 2000 (Inovio Pharmaceuticals, CEPI);DNA plasmid encoding S protein delivered by electroporation with CELLECTRA^®^ 2000)/intradermal DNA vaccine/18 years and older	NCT04336410 (ph 1 SUA, ph 2 USA)/July 2021
INO-4800 +electroporation using CELLECTRA 2000 (International Vaccine Institute, CEPI, Inovio Pharmaceuticals)/19 to 64 Year	NCT04447781 (ph1-2, Republic of Korea)/February 2022
GX-19 (Genexine,Inc); DNA vaccine expressing SARS-CoV-2 S-protein antigen/18 to 50 Years	NCT04445389 (ph1-2, Republic of Korea/March, 2021

Resources: U.S. National Library of Medicine https://clinicaltrials.gov International Clinical Trials Registry Platform (ICTRP)**/** https://www.isrctn.com/.

**Table 4:** Drags targeting SARS-CoV-2 and host immunity included in the article.

**Therapeutic target**	**Drug and article reference**	**Drugs related studies**
**Receptor recognition (ACE2, CD147 receptors)**	Human recombinant soluble form of ACE2 (Khan et al., 2017; Monteil et al., 2020; Monteil et al., 2020).	NCT04335136, NCT04375046 NCT04287686 (withdrawn)
	Meplazumab (mAb antiCD147 receptor) (Wang K. et al., 2020)	NCT04275245
**Clathrin-mediated endocytosis**	Hydroxychloroquine (Hq) sulphate (Picot et al., 1993; Jang et al., 2006; Bender et al., 2020) and Chloroquine phosphate (Savarino et al., 2003; Vincent et al., 2005) *multiple targets: clathrin-mediated endocytosis, endosomal Ph, TLR7/8, IFN response, and proinflammatory cytokines release) Controversial results: (Borba et al., 2020; Lane et al., 2020; Shamshirian et al., 2020; Tu et al., 2020)	-ongoing studies NCT04315948, DisCoVeRy trial, NCT04304053 ChiCTR2000029803, NCT04334148,ChiCTR2000029609**^-^** **-**discontinued study: Hq arm of Solidarity trial -withdrawn: NCT04347512, NCT04371926
	Baricitinib (Richardson et al., 2020; Stebbing et al., 2020) Ruxolitinib (Gaspari et al., 2020; Yeleswaram et al., 2020)	NCT04320277, NCT04401579 NCT04362137
**Endosomal fusion**	Umifenovir (Deng et al., 2020; Lian et al., 2020)	IRCT20151227025726N15
	Camostat mesylate (Kawase et al., 2012; Yamamoto et al., 2016; Hoffmann et al., 2020b) * multiple targets: TMPRSS2, endosomal fusion	NCT04321096
**Viral synthesis**	Protease inhibitors: lopinavir/darunavir/ritonavir (3CLpro, PLpro)	RECOVERY trial, NCT04251871, NCT04255017, ChiCTR2000029539 NCT04252274, NCT04295551, ChiCTR2000029308, NCT04252274
	Remdesivir * (Sheahan et al., 2017; Agostini et al., 2018; Beigel et al., 2020; Goldman et al., 2020; Grein et al., 2020; Wang M. et al., 2020; Wu et al., 2020a; Wang Y. et al., 2020) *multiple targets: viral autophagy, mTORC1, Nsp12-RdRp, replicase/transcription complex –RTC	Trial ACTT-NCT04280705 si ACTT-II, NCT04292899 NCT04257656, SIMPLE trial, WHO Solidarity trial, DisCoVeRy trial
	Favipiravir/target: Nsp12-RdRp) (Coomes and Haghbayan, 2020)	ChiCTR2000030254, ChiCTR2000029544 ChiCTR2000029600
**Cytokine response, Th1 response**	IFN administration (Hung et al., 2020) Controversial results (Gandhi, 2020)	See **Table 1**
	Ruxolitinib (anti-Janus kinase inhibitor) (Gaspari et al., 2020; Yeleswaram et al., 2020)	NCT04362137
**IL-6 production**	Tocilizumab (Xu et al., 2020). (Anti IL-6 mAb)	NCT04317092, NCT04315480 COVACTA, NCT04320615
	Sarilumab and siltuximab (Anti IL-6 mAb)	NCT04324073, NCT04315298, NCT04329650
**VEGFA**	Bevacizumab (anti-VEGFA mAb)	NCT04275414
**GM-CSF**	Gimsilumab (Zhou Y. et al., 2020) (anti GM-CSF mAb)	BREATH clinical trial
**Inflammatory response**	Corticosteroids (Horby et al., 2020; Wu et al., 2020b).	UK RECOVERY trial NCT04381936
**Immuno-modulatoy activity**	Mesenchymal stem cells (Shi et al., 2018; Leng et al., 2020)/ *multiple targets:inflammatory cytokines, Th2 response, regeneration of damaged cell	See **Table 2**
	Thalidomide	NCT04273529, NCT04273581
	Fingolimod	NCT04280588
**Neutralizing antibodies**	Convalescent plasma (Rogers et al., 2020; Zhou and Zhao, 2020)	NCT04381936, NCT04373460, ISRCTN50189673, NCT04348656
	mAb (Sui et al., 2004; Elshabrawy et al., 2012); neutralizing IgG1 monoclonal antibody (mAb) directed against the spike protein of SARS-CoV-2 (LY-CoV555) JS016	NCT04427501, NCT04441918
	-cross-reactive neutralizing antibodies against SARS-CoV-2 RBD specific epitopes (Lan et al., 2020; Wang C. et al., 2020; Yuan et al., 2020)	See **Table 3**

The production of a SARS-CoV-2 vaccine has met major obstacles since the beginning such as the absence of clear data on the immune response elicited by SARS-CoV-2 and the notable side effects observed in SARS-CoV and MERS vaccine candidates (Bolles et al., 2011; Tseng et al., 2012; Agrawal et al., 2016). Additionally, a decreasing confidence of the general population in the vaccination effort can become a major obstacle for the development of a efficient vaccination programme.

Listed below are the types of vaccines against COVID-19 which have passed preclinical testing and have entered the clinical setting:

- *Vector-Based Vaccines* induce a predominant cytotoxic T lymphocyte (CTL) response, and display a high efficiency. Their response is additionally amplified through the adjuvant vector. However, the “pre-existing’’ immunity to the vector could influence the development of an immune response and represents a disadvantage (ex: Ad5-nCoV by CanSino, ChAdOx1 nCoV-19 by Oxford). Recently, the researchers from Oxford Vaccine Trial Group reported the preliminary results of a phase I/II randomized controlled trial on ChAdOx1 nCoV-19 vaccine. This data confirms its immunogenicity and safety as well as its ability to induce humoral and cellular immunity, paving its entry into phase II/III studies (Folegatti et al., 2020).
- *Nucleic Acid Vaccine* also trigger a predominant CTL response. The associated immune response is satisfactory, the manufacturing process is simple but there could be obstacles regarding vaccine delivery. Multiple transport systems have been tested such as lipid nanoparticles, plasmids or vectors. The administration of certain vaccines is performed through electroporation (ex: INO-4800 by Inovio, Mrna-1273 by Moderna).
- *Inactivated Vaccines* induce a protective B cell response, yet this appears to be modest and requires booster shots. These vaccines are safe but expensive, difficult to produce in large quantities and require a well controlled environment during their manufacture (ex. Coronavac by Sinovac).
- *Protein-Based Subunit Vaccines* trigger a humoral response by using peptide fragments of the S protein (S1 or RBD subunit). In order to obtain an adequate immune response, these peptides need to be coupled to adjuvants which in turn require an additional follow-up for potential side-effects (ex: NVX-CoV2373 by Novavax).
- *Non-Specific Vaccines*. BCG, the vaccine against Mycobacterium tuberculosis is the non-specific vaccine which has received most attention in the SARS-CoV-2 infection due to its ability to boost the immunity against SARS-CoV-2. BCG is currently investigated in multiple randomized trials across the globe, many of which have reached phase 3 or 4 and have begun the recruitment of volunteers. Clinical trials will evaluate the non-specific immune response elicited by BCG and its benefit on lowering the incidence of COVID-19 in the vaccinated general population (NCT04328441), in children (NCT04327206) or in health care workers exposed to SARS-CoV-2 (NCT04379336, NCT04327206, NCT04348370, NCT04327206, NCT04348370). Of the vaccines currently undergoing preclinical studies AAVCOVID-by AveXis is a distinctive gene-based vaccine candidate which deserves a separate mention. AAVCOVID is an experimental gene-vaccine which expresses SARS-nCoV-2 Spike antigens delivered through a vector already used in ophthalmology (adeno-associated viral vectors/AAV), which differs from the human adenoviral vector. The AAV technology has already been used in gene therapy and is more advantageous due to its availability, safety profile and immunogenic response without the risk of pre-existing immunity. AAV vaccines are responsible for strong antibody responses and high T cell memory responses.

On a side note, while a highly immunogenic and safe vaccine is the most desirable strategy to prevent the pandemic extension, one should not forget that the immune response to the vaccine can be variable, short or incomplete and that the vulnerable groups do not always respond to antigenic stimulation. Large studies are therefore needed to verify the safety and efficiency and further efforts are needed to boost the confidence of the population in vaccination practices and in the healthcare system.

## Conclusion

The current therapeutic options in COVID-19 combine a diverse armamentarium of drugs targeting both the virus and the immune response to avert this unexpected and extremely dangerous pandemic (**Table 4**). The number of experimental molecules proposed against SARS-CoV-2 is continually rising in an attempt to offer short-term solutions, especially for patients with a severe disease (Tu et al., 2020). As a result, it has become clearer that these options should be weighted with responsibility and should be tailored to the complex pathogenic context of COVID-19. Thus, molecules which could be beneficial in a certain stage of the disease could be devastating in another. Hence, the best treatment option appears to be a molecule which would restrict viral entry and to impede an unpredictable immune response. On the long-term, this would be ensured only by the production of a vaccine, while on the short-term the best treatment option requires further in-depth studies on the cellular invasion and immune dysregulation.

Despite the barriers which impede the development of a rapid treatment, current therapeutic approaches represent a stepping stone for the handling of future epidemics. The SARS-CoV-2 infection proved once again that viruses leave a very short window of opportunity, far too short for adequate antiviral options. This unfortunate prospect is now even more relevant and further similar scenarios can occur at any time with other types of pathogens, potentially reaching a global scale as in the case of SARS-CoV-2. Hence, this also serves as a brutal warning that our current therapeutic agents are insufficient against infectious agents and that non-etiologic medications could potentially play an important role in the future.

## Author Contributions

SI and DI contributed equally to the acquisition, analysis, and critical revision of the article; the authors have given their consent for the publication and agree to be responsible for the accuracy and integrity of the article.

## Conflict of Interest

The authors declare that the research was conducted in the absence of any commercial or financial relationships that could be construed as a potential conflict of interest.
